# Medical College Notes

**Published:** 1892-01

**Authors:** 


### Medical College Notes.

COLUMBUS, O., December 3, 1891.

PURSUANT to call issued by the Cincinnati College of Medicine and Surgery for a delegated convention of the medical colleges of the State of Ohio, to be held at Columbus on the date above mentioned, representatives of the following faculties were present, viz.:— Starling Medical College, Toledo Medical College, Pulte Medical College, Columbus Medical College, Medical Department of the National Normal University, College of Physicians and Surgeons of Columbus, Woman's Medical College of Cincinnati, and the Cincinnati College of Medicine and Surgery.

On motion, Dr. STARLING LOVING was elected Chairman, and Dr. CHARLES A. L. REED, Secretary.

On motion of Dr. C. E. WALTON, representatives of the Physio-Medical Society of Ohio were admitted to a vote in the convention.

Dr. CHARLES A. L. REED presented the following :

*Resolved*, By the medical colleges of Ohio, in convention assembled, that the Legislature be and is hereby requested to enact a law which shall embody the following features, viz. :

1. The creation of a board or boards of medical examiners, in the composition of which equitable and just representation shall be accorded to the various recognized denominations of medical practice.

2. The examination of all candidates for the practice of medicine holding diplomas hereafter issued by medical colleges which shall be deemed in good standing by the board.

3. Exemptions from examination to extend only to those who at the time of the enactment of this law shall be recognized as legal practitioners within the meaning of existing statutes ; but all legal practitioners shall be required to register.

4. A penal clause which shall secure the enforcement of the foregoing provisions.

Dr. C. E. WALTON, on behalf of the Legislative Committee of Cincinnati, presented the registration law approved and promulgated by that committee.

On motion by Dr. SHOCKEY, the resolutions presented by Dr. Reed were approved.

On motion by Dr. KINSMAN, the Secretary was directed to forward transcripts of these proceedings to each local medical society in Ohio, and to the medical press.

On motion by Dr. Scoville, a committee was appointed to confer with the Legislative Committee of Cincinnati for the purpose of securing such changes in the bill proposed by that committee as to make it conform to the resolutions adopted by this convention.

The chair appointed as such committee: Drs. S. S. Scoville, T. C. Hoover, G. W. Mayhugh, and Chas. A. L. Reed. Adjourned.

---

UNIVERSITY OF BUFFALO, DEPARTMENT OF PHARMACY.—The laboratories of this school are being refurnished and new apparatus supplied. There are bright prospects for a large first year class, a considerable proportion of the new students coming from out of the city. Prof. Long will devote more time to toxicology than previously. Prof. Wende will make no radical change in the microscopic course, but the junior work will be somewhat increased. Dr. Benedict will devote more time to plant classification than last year; otherwise the botany course will remain practically unaltered.

A material increase in the hours of study for the juniors will be made. Arrangements have just been completed for the purchase of a set of 150 different pharmacopeial drugs. These will be added to the already large collection of botanical and pharmacological specimens. Although the college does not contract to furnish positions to students, it has been found that students with previous experience in drug stores have had no difficulty in obtaining situations. The members of the faculty render all possible assistance in this regard.—*Bulletin of Pharmacy, October, 1891.*

---

MRS. C. P. HUNTINGTON has given the directors of the Post-Graduate Medical School $3,000, a sum sufficient to defray the expenses of the lying-in department for one year. Prof. von Ramdohr will have charge of this department, at 543 East 13th street, where instruction in obstetrics will be given to graduates in medicine only.

